# Unilateral Hypertrophy of the Tensor Fascia Lata

**DOI:** 10.5334/jbsr.3462

**Published:** 2024-03-21

**Authors:** Carla Pereira, Aman Toukouki, William Kurth

**Affiliations:** 1 Avenue de l'Hôpital 1,4000 Liège, Chu Liège, Belgium; 2 Avenue de l'Hôpital 1,4000 Liège, Chu Liège, Belgium; 3 Avenue de l'Hôpital 1,4000 Liège, Chu Liège, Belgium

**Keywords:** Tensor fascia lata, gluteus tendinopathy, ultrasound, MRI

## Abstract

A 57-year-old woman presented with right hip pain. The initial diagnosis was an inflammatory tendinopathy of the gluteus medius without signs of rupture, and the patient underwent an ultrasound-guided corticosteroid injection. However, 1 month later, she presented with a painful swelling on the anterior-external aspect of the right hip/thigh, with a clinical suspicion of malignancy. Magnetic resonance imaging (MRI) scan and ultrasound confirmed the diagnosis of hypertrophy of the tensor fascia lata (TFL) muscle . It is included in the differential diagnosis of soft tissue masses of the anterolateral proximal part of the thigh. The etiology is likely to be associated with gluteal muscle dysfunction.

*Teaching point:* Unilateral hypertrophy of the fascia lata consists of an association with hypertrophy of the tensor fascia lata muscle and pathology of the minimus and medius gluteus muscles.

## Case Report

A 57-year-old woman presented with right hip pain in the supratrochanteric area. Ultrasound suggested an inflammatory tendinopathy of the gluteus medius without signs of rupture. An ultrasound-guided corticosteroid injection was performed without sign of immediate complication.

One month later, the patient complained of the appearance of a painful mass at the anterior-external face of the right proximal crural region. The ultrasound examination demonstrated clear asymmetry due to hypertrophy of the right tensor fascia lata (TFL) muscle and without a focal intramuscular mass lesion.

**Figure 1:**
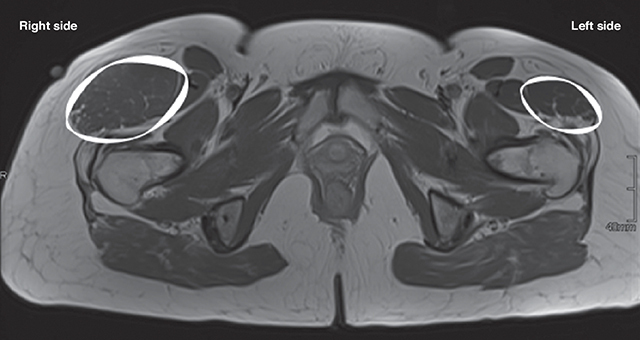
The sequence axial T1 shows the hypertrophic tensor fascia lata (white) on the right (surface: 1.855 mm^2^) compared to the left side (surface: 789 mm^2^).

**Figure 2:**
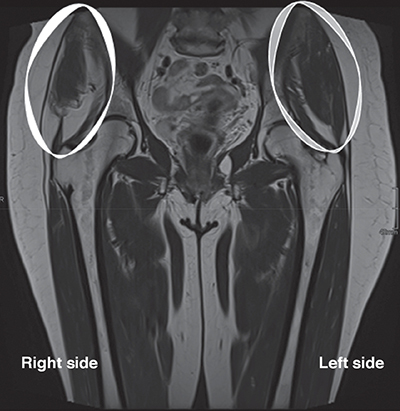
Coronal pelvic MRI demonstrates evidence of significant fatty atrophy of the right gluteus muscles compared to the left side.

Three months later, a magnetic resonance imaging (MRI) of the pelvis was performed for the precise characterization of this pseudotumor. A striking asymmetry of dimensions of the TFL muscles was observed (Figure 1, circle). On all MRI sequences, including gadolinium-enhanced sequences, there was no muscle abnormality, aponeurotic lesion, or mass lesion. Fatty atrophy with partial tears of the minimus and medius gluteus muscles with infiltration of the iliotibial band were found (Figure 2, circle).

The diagnosis of an isolated pseudotumor of the TFL muscle was retained.

## Comment

A pseudotumor of the TFL is defined as the association of hypertrophy of the TFL and a pathology of the gluteus minimus and medius muscles [1, 3]. TFL hypertrophy is a rare benign clinical entity with a painful mass on the anterolateral thigh [1–3]. The differential diagnosis includes malignancy, as sarcoma. MRI and ultrasound (US) are contributive to distinguish malignant from benign and pseudotumors.

Hypertrophy of TFL can have a variety of presentations, including a painful mass on the anterolateral hip/groin often with symptoms that may mimic chronic lumbosacral radiculopathy [1].

It is hypothesized that the etiology of TFL hypertrophy may be mechanical versus denervation.

In the case described, it is most likely a compensatory hypertrophy following gluteus tendon tears and atrophy of gluteus muscles. This suggests that the TFL may have a compensatory role in patients with weakness of hip abduction in gluteal pathology.

Compensatory hypertrophy may also occur following hip osteoarthritis, anterior cruciate ligament tears, prior hip surgery (i.e., hip replacement), and spine surgery. There should be awareness that postoperative patients may develop TFL hypertrophy [1].

MRI is the imaging modality of choice for confirming TFL hypertrophy and thus avoids unnecessary biopsy or surgery [2]. Treatment consists in an association of the regularly strengthening of the abductor muscles and of clinical follow-up. Botulinum toxin injections may be interesting to improve persistent TFL symptoms in selected patients [1].

## References

[r1] Shields LBE, Iyer V, Bhupalam RC, Zhang YP, Shields CB. Hypertrophy of the tensor fascia lata: A pseudotumor due to lumbar radiculopathy. Surg Neurol Int. 2021;12:522. 34754572 10.25259/SNI_857_2021 PMC8571211

[r2] Ilaslan H, Wenger DE, Shives TC, Unni KK. Unilateral hypertrophy of tensor fascia lata: A soft tissue tumor simulator. Skeletal Radiol. 2003 Nov; 32(11):628–632. DOI: 10.1007/s00256-003-0687-0. Epub 2003 Sep 30. PMID: . 14586575

[r3] De Clercq C, Jans L, Verstraete K. Bilateral hypertrophy of the m. Tensor fascia latae. J Belg Soc Radiol. 9, 2022 May;106(1):44. DOI: 10.5334/jbsr.2724. PMID: ; PMCID: . 35647483 PMC9104491

